# Southern hemisphere forced millennial scale Indian summer monsoon variability during the late Pleistocene

**DOI:** 10.1038/s41598-022-14010-6

**Published:** 2022-06-16

**Authors:** Shraddha T. Band, M. G. Yadava, Nikita Kaushal, M. Midhun, Kaustubh Thirumalai, Timmy Francis, Amzad Laskar, R. Ramesh, Gideon M. Henderson, A. C. Narayana

**Affiliations:** 1 Geosciences Division, Physical Research Laboratory, Ahmedabad, India; 2 Department of Geosciences, National Taiwan University, Taipei, Taiwan; 3 Department of Earth Sciences, University of Oxford, Oxford, UK; 4 Department of Atmospheric Sciences, School of Marine Sciences, Cochin University of Science and Technology, Kochi, India; 5 Department of Geosciences, University of Arizona, 1040 E. 4th Street, Tucson, AZ 85721 USA; 6 National Centre for Medium Range Weather Forecasting, Noida, India; 7 Centre for Earth, Ocean and Atmospheric Sciences, University of Hyderabad, Hyderabad, 560 046 India; 8 Present Address: Department of Earth Sciences, ETH Zurich, Zürich, Switzerland

**Keywords:** Climate sciences, Environmental sciences, Hydrology

## Abstract

Peninsular India hosts the initial rain-down of the Indian Summer Monsoon (ISM) after which winds travel further east inwards into Asia. Stalagmite oxygen isotope composition from this region, such as those from Belum Cave, preserve the vital signals of the past ISM variability. These archives experience a single wet season with a single dominant moisture source annually. Here we present high-resolution δ^18^O, δ^13^C and trace element (Mg/Ca, Sr/Ca, Ba/Ca, Mn/Ca) time series from a Belum Cave stalagmite spanning glacial MIS-6 (from ~ 183 to ~ 175 kyr) and interglacial substages MIS-5c-5a (~ 104 kyr to ~ 82 kyr). With most paleomonsoon reconstructions reporting coherent evolution of northern hemisphere summer insolation and ISM variability on orbital timescale, we focus on understanding the mechanisms behind millennial scale variability. Finding that the two are decoupled over millennial timescales, we address the role of the Southern Hemisphere processes in modulating monsoon strength as a part of the Hadley circulation. We identify several strong and weak episodes of ISM intensity during 104–82 kyr. Some of the weak episodes correspond to warming in the southern hemisphere associated with weak cross-equatorial winds. We show that during the MIS-5 substages, ISM strength gradually declined with millennial scale variability linked to Southern Hemisphere temperature changes which in turn modulate the strength of the Mascarene High.

## Introduction

**Figure 1:**
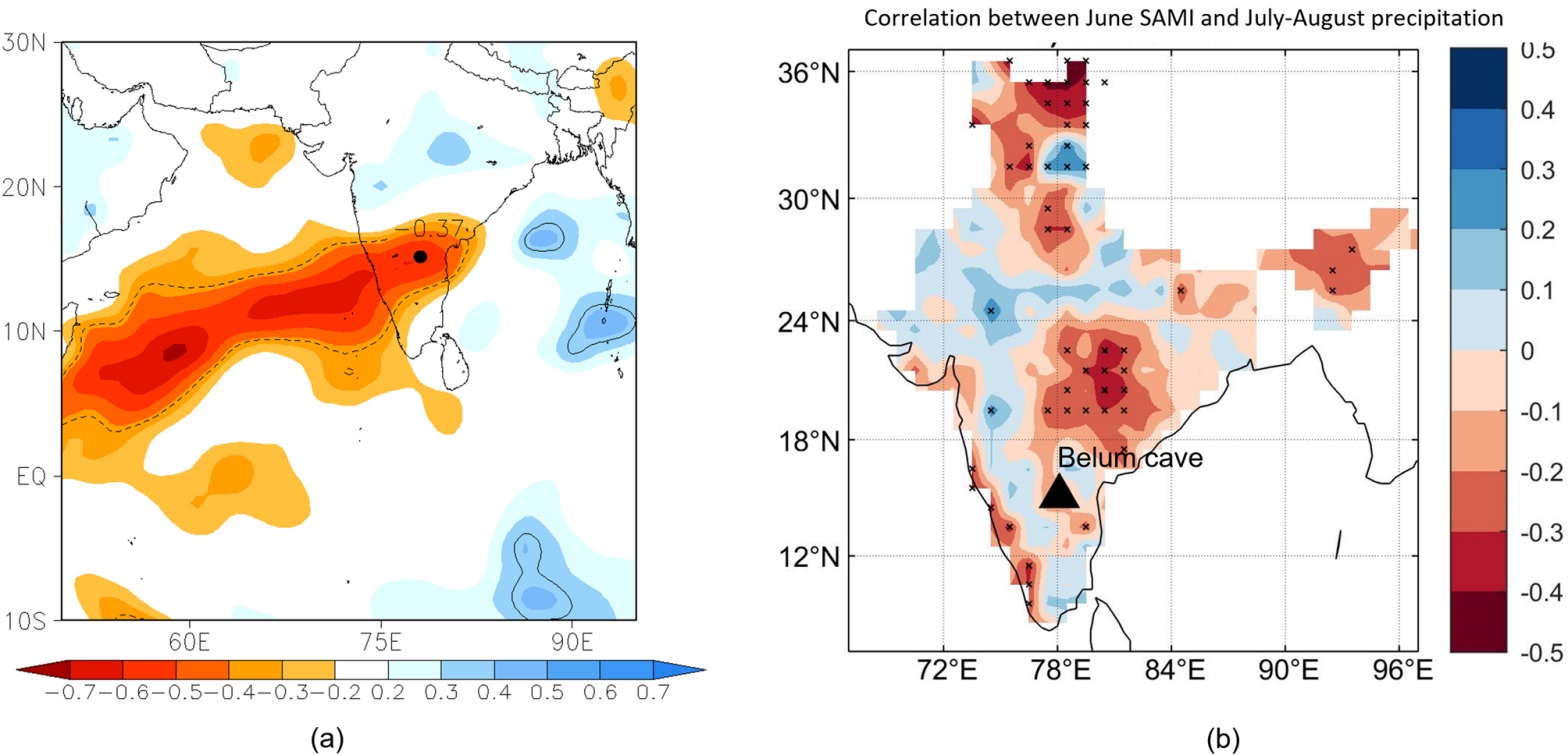
(**a**) Correlation coefficient between IsoGSM model simulated JJAS average rainfall δ^18^O at Belum Cave (Model resolution is ~ 200 × 200 km) and simulated JJAS average rainfall over surrounding grids^15^. Correlation (= − 0.37) with significance level *p* = 0.05 is marked by black dashed contour. (**b**) Correlation between July–August precipitation and June SAMI (Southern Hemisphere Annular Mode Index) for the period 1957–2018.

The Indian Summer Monsoon (ISM) an integral part of the Asian Summer Monsoon (ASM) system is an annual climate manifestation of the seasonal reversal of cross-equatorial winds, which result in widespread precipitation over the Indian subcontinent during the months of June–September^1–3^. Proxy records of ISM strength from peninsular India are important as they record a signal of synoptic-scale monsoon circulation^4^ and are good indicators of overall ISM strength, in addition to its core variability, across different timescales (annual to orbital variability). A few speleothem-based ISM reconstructions exist from the ISM region^5–13^. However, the δ^18^O signatures of these speleothem records are thought to have been derived from different moisture sources or upstream changes in the δ^18^O of precipitation in the ISM domain (rather than regional rainfall amount variability) thus complicating interpretations^14^. Belum Cave (15° 6’ N, 78° 6’ E, 367 m above m.s.l. Fig. 1) located in Peninsular India receives the initial spells of ISM rainfall, as monsoon winds transport moisture from the Indian Ocean and Arabian Sea to peninsular India before moving further eastward over the Bay of Bengal and into Asia. The Belum Cave stalagmite (Fig. 1), reported in this study is composed of calcite δ^18^O and provides a record of glacial MIS-6 and interglacial MIS-5 ISM variability with reasonable age control (average age uncertainty of 2000 years). Trace element ratios including Mg/Ca and Sr/Ca in the Belum stalagmite provide independent and valuable information that supports the interpretation of the δ^18^O record.

ISM is strongly linked to several ocean-atmospheric processes operating on various timescales. On inter-annual to decadal timescale ISM intensity is influenced by El Niño–Southern Oscillation (ENSO) in the Equatorial Pacific^16–18^, Indian Ocean Dipole^19,20^, Northern Atlantic Oscillations^21^, and the Southern Annular Mode (SAM)^22,23^. On longer timescales, ISM is modulated by variations in orbital parameters^24^. Recent findings also associate strength of ISM and Agulhas Current variability based on frontal shifts in the Southern Ocean^25^. Whereas many studies focused on teleconnections between abrupt high latitude North Atlantic climate events (Heinrich events, Dansgaard-Oeschger oscillations, Younger Dryas, Bølling-Allerød period) and ISM variability^26–30^, few examine linkages with Southern Hemisphere climate variability, which has also been outlined as a potential modulator^25,30–32^ of ISM variability. In this study, we aim to advance our understanding of the Southern Indian Ocean in modulating ISMR variability^25,30–33^.

## Results

To identify the moisture sources contributing rainfall to the cave site during the wet season (June to October), we carried out Lagrangian back trajectory analysis using HYSPLIT model^34^ with NCEP Reanalysis-1^35^ as the input to the model. We chose all the days with daily rainfall amount above 2 mm during a 10-year period (1998–2007). During the southwest monsoon period of JJAS (June, July, August, September), trajectories suggest that the Arabian Sea is the major source of moisture, whereas, during October, it is a mixture of Arabian Sea and the Bay of Bengal sourced moisture (SI-Fig. 1). A major portion of annual rainfall, however, is still obtained from the Arabian Sea moisture, and the vapor reaching the cave is the remnant of the rainfall that occurred in the north–south oriented rainfall belt across the Western Ghats^36^. Hence, the rainfall isotopic composition over Belum Cave is likely to reflect the degree of rainout over the Western Ghats region and evaporative loss of rain-forming vapor of low to mid convective strength cloud top during summer monsoon^36^. A significant negative correlation between δ^18^O of Belum rain and amount of rain recorded by nearest meteorological station was shown in a previous study by Yadava et al. 2016^9^ (May to January, 2011 CE; n = 20). Results from an IsoGSM model run using the spectral nudging technique^15,37–39^ further show that the inter-annual variation of the ISM rainfall δ^18^O values at Belum Cave are well correlated with ISM rainfall over the Arabian Sea and Western Ghat region (Fig. 1). This strengthens our hypothesis that Belum Cave rainfall isotopic composition is mainly controlled by the rainfall amount over the Arabian Sea and Western Ghat region and hence δ^18^O of our speleothem, BLM-1, is a proxy for past monsoon strength which is in turn related to the strength of the larger Hadley circulation.

As the aim of the present study is to address linkages between ISM and Southern Hemisphere climate variability, we correlated one of the climate components of extra-tropical Southern Hemisphere Annular Mode Index (SAMI) with July–August ISM rainfall (Fig. 1b; daily gridded rainfall data (1° × 1°) between 1957 and 2018 was taken from the Indian Meteorological Department^40^). Viswambharan et al. 2012^22^ demonstrated the existence of a significant relationship between SAMI and Northern Hemisphere monsoon circulation patterns. The SAMI which is based on the zonal pressure difference between the latitudes of 40° S and 65° S, was estimated for the same period of 61 years (1957–2018)^41^. The high and low SAMI years were defined according to Viswambharan et al. 2012^22^ where they correspond to years above and below one standard deviation of the SAMI distribution. We correlate June SAMI and July -August ISMR (active phase of monsoon) for years 1957–2018 and observe a significant negative correlation between June SAMI and July–August ISMR for southern western and central parts of peninsular India.

**Figure 2:**
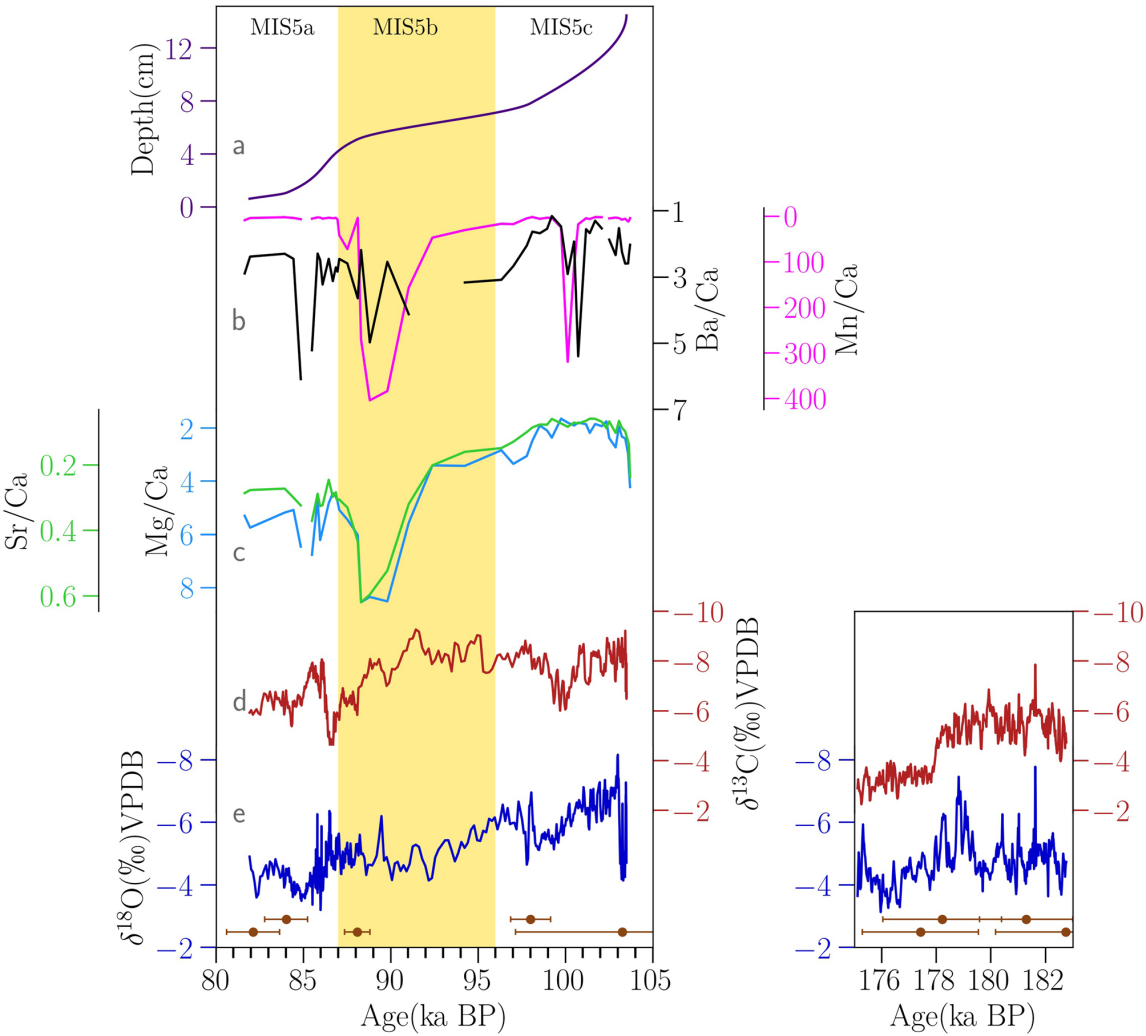
(**a**) Growth rate (cm/kyr) (**b**) Mn/Ca (pink) and Ba/Ca (black) (**c**) Sr/Ca (green) and Mg/Ca (light blue) trace element ratios are shown for the MIS-5 substages. (**d**) δ^13^C (red) and (**e**) δ^18^O (blue) profiles are for the Belum Cave stalagmite (present work). The ^230^Th ages with 2σ are shown as filled circles (orange) and the stadial MIS-5b (yellow).

The oldest part of the stalagmite (section-1, SI Fig. S2) grew during the glacial stage MIS-6, from 182 to 175 kyr, with ages having an average error of ~ 2000 years in this part. Growth rate during this period was 1.8 cm/kyr prior to 176 kyr, after which it increased to 2 cm/kyr. Oxygen and carbon isotopic values, between 182 to 178 kyr, show co-variability during this period and have an average value of ~ − 5‰ (Fig. 2,2). Subsequently, between 178 and 175 kyr, oxygen isotopic values are found to be higher by 1‰ and the carbon isotopic values are higher by 2‰.

Following this, the stalagmite has a ~ 5 cm portion composed of detrital rich material (Section-2, SI Fig. S2)”. This part of the stalagmite covers the transition period from glacial MIS 6 to interglacial MIS 5. ‘Clean’ calcite began to grow again during interglacial MIS-5 from 103 to 82 kyr, with an average age error of ~ 1000 years (Section-3, SI Fig. S2”). The growth rate between 97 and 88 kyr (MIS-5b) was steady at 0.2 cm/kyr (Fig. 2a). This was preceded and succeeded by two peaks of the rapid growth of 1.5 cm/kyr. The oxygen isotopic values get progressively higher from 103 to 82 kyr by 2‰, starting at − 7‰ and eventually reaching glacial values of − 5‰ (Fig. 2e). The carbon isotopic values are consistently lower than the glacial values at ~ − 8‰ and increase only after 90 kyr to ~ − 7‰ (Fig. 2d). The average difference between the oxygen and carbon isotopic values, in the interglacial MIS-5, is ~ 2‰. Trace element ratios have been measured for the MIS-5 portion of the stalagmite (MIS 5b and c) with resolution of ~ 500 years. Mg/Ca and Sr/Ca time series show high correlation (r^2^ = 0.95). Along with Ba/Ca show a steady increase from 103 to 82 kyr with a sharp excursion towards higher values at 88 kyr. This sharp excursion is also seen in Mn/Ca values.

## Discussion

This study provides some of the earliest δ^18^O measurements for glacial/interglacial periods from the peninsular Indian region during the late Pleistocene period^42^. Presently, the cave site receives moisture predominantly from the Arabian Sea during a single monsoon season extending from June to September. The 3‰ difference in δ^18^O values seen between the peak during the interglacial period and the more glacial conditions must reflect the combined effects of changes in: (1) the distance of moisture source from cave site reflecting shift in atmospheric circulation, (2) moisture composition in the source region, (3) the amount effect resulting from modification of upstream and local rainfall amounts, and 4) changes in internal cave conditions^43^.

**Figure 3:**
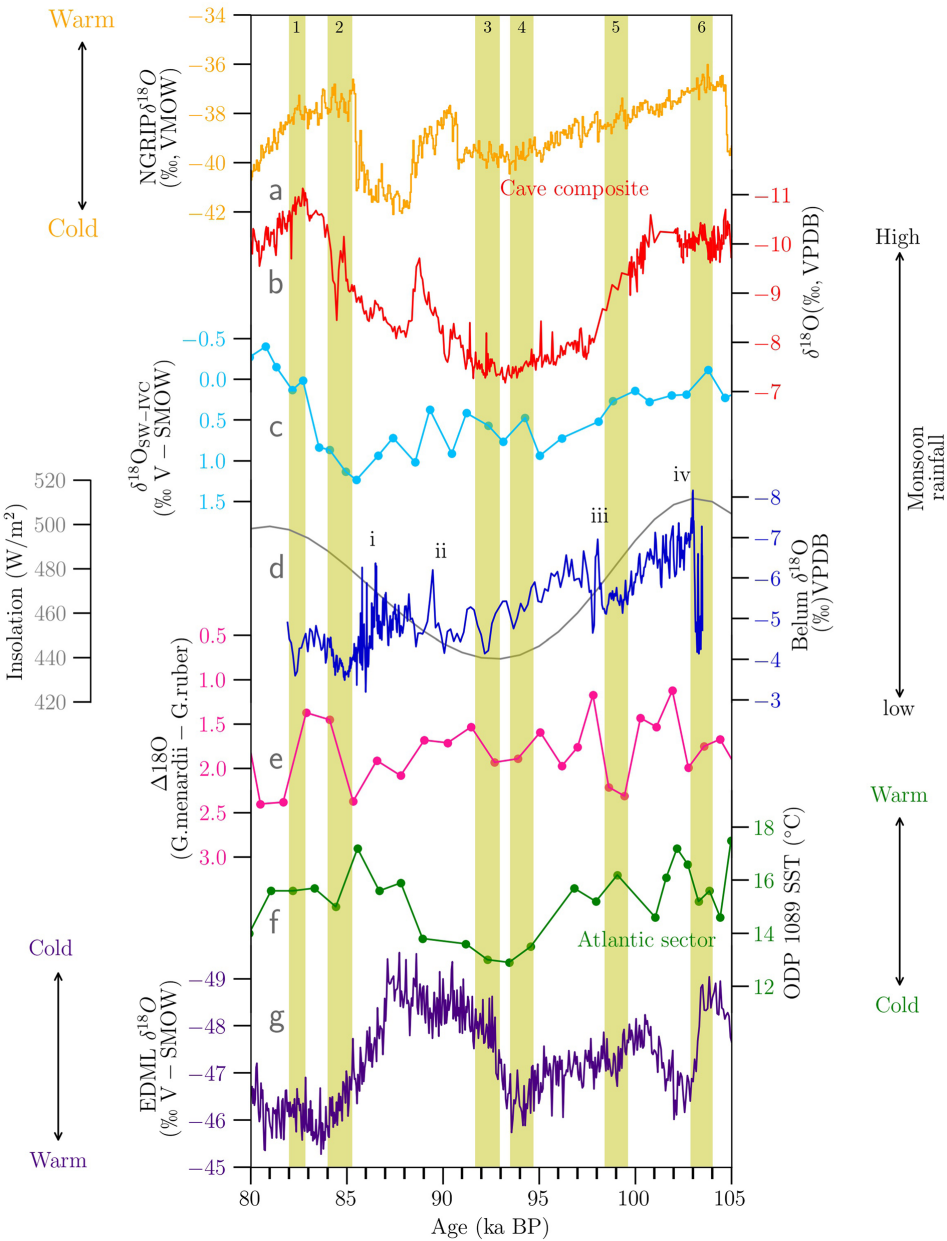
(**a**) NGRIP ice core δ^18^O record^48^ (**b**) Cave composite δ^18^O record based on Chinese speleothems^24^ (**c**) ice volume corrected δ^18^O_sw-ivc_ record from site SO-188-17,286-1^49^, northern Bay of Bengal (**d**) Belum Cave δ^18^O record (Present study) and insolation at 30° N (dark grey) (**e**) difference between δ^18^O of surface (*G.ruber*) and thermocline dwelling (*G.menardii*) foraminifera from tropical Southern Hemisphere (core BP13/A)^50^. (**f**) Radiolarian based SST reconstruction from the core site ODP 1089 northern subantarctic in Atlantic sector^51^. (**f**) EDML δ^18^O record^52^. The shaded yellow areas numbered 1–6 represent episodes of weaker monsoon observed in our record and i-iv correspond to strong monsoon events. The locations of the core sites are shown in SI Fig. S3.

Section 3 (103–82 kyr BP) of the stalagmite addresses the variability in ISM during the interstadials 5c-5a, when gradual cooling was observed globally before entering full glacial conditions at 74 kyr^44^ (Fig. 3a). In our reconstruction, gradual increase in oxygen isotopic values of − 8‰ to ~ − 4‰, indicates a corresponding decrease in ISM intensity from MIS-5c (103 kyr) to 5a (82 kyr). There is growing understanding that speleothem oxygen isotopes reflect larger circulation changes. In contrast, changes in carbon isotopes, trace element ratios and growth rates are more likely to reflect local karstic (environmental), climatic, and hydrological changes^45^. The strong correlation seen between Mg/Ca and Sr/Ca measured in the MIS-5 growth portion of the stalagmite suggests that they are controlled by similar processes. Since the analysis material for trace element measurements was sampled distinctly and at a different resolution to stable isotopes, it is not possible to make a one-to-one comparison with stable isotopes. However, the long-term trend suggests that the trace element ratios progressively increased from MIS-5c toward MIS-5a, mirroring a similar trend in δ^18^O values (Fig. 2). A similar pattern is not observed in case the of δ^13^C. The correlation between Mg/Ca, Sr/Ca and Ba/Ca (Fig. 2), reflects a common source^46,47^ rather than the impact of a partitioning mechanism such as Prior Carbonate Precipitation (PCP) which is caused by degassing prior to speleothem formation, and would be reflected in the δ^13^C values as well. In this case, the long-term increasing trend in Mg/Ca and Sr/Ca values more likely suggests progressively longer water residence times and drier conditions.

However, we stress that further monitoring work is required in this cave region. The increased detrital deposition and hiatus periods (part of  section-2, SI Fig S2) or lack of further growth during transitions between the two periods (representing  sections 1 and 3, SI Fig S2) suggests that a different region of this vast cave network may have to be investigated for suitable samples. Higher resolution trace element ratio analysis is required to confirm interpretations of local climatic changes.

We compared our records with NGRIP δ^18^O timeseries and observed that between 103 and 91 kyr BP (Fig. 3a), ISM and North Atlantic climate change trends are coherent. However, between 91 and 83 kyr BP, the relationship in the δ^18^O timeseries changes. In our Belum cave record, this period from 91 to 83 kyr (transition MIS 5.2/5.1 transition) doesn’t follow the decrease and subsequent increase in δ^18^O timeseries seen in the NGRIP and EASM cave composite records. However, this period manifests as a distinct anomaly in our trace element record and carbon isotopic records. It appears that this event maybe masked by the millennial scale southern hemisphere driven climate changes in oxygen isotope record but are nevertheless reflected in proxies recording regional hydroclimatic change. Recent studies also point towards dissociation between East Asian Summer Monsoon reconstructions (EASM) and ISM variability over the Pleistocene^53,54^. To address this we compared the cave composite record from China^55^ with our record (Fig. 3b). Except for the Weak Monsoon interval 2 (yellow shaded area), we could not find any synchroneity between the inferred ISM and EASM variability. Lauterbach et al. 2020^49^ reconstructed SSTs from sediment core SO 188‐17,286‐1 raised from the northern Bay of Bengal to reconstruct ISM variability based on ice-volume corrected δ^18^Osw‐ivc of *G.ruber.* In this comparison, we observe that stronger monsoon episodes in both records are concurrent (Fig. 3c)—an inference that suggests the observed linkage of ISM depressions to Bay of Bengal temperatures^4^ could have operated on millennial timescales as well.

To understand the possible linkage between Southern Hemisphere climate variability and ISM, we compared our record with Antarctic ice core (EDML record) δ^18^O timeseries, Agulhas SST variability (ODP 1089), and an upwelling record from the tropical Southern Hemisphere (core BP 13/A). We identify six episodes of weaker monsoon and five episodes of stronger monsoon intervals (Fig. 3). Based on previously detailed mecahnisms^25,50^, we expect that warm (cold) high-latitude Southern Hemisphere temperatures will correspond to weak (strong) ISM variability^25,50,56^. We first compare our record with the southernmost high-resolution EDML δ^18^O timeseries^52^ from the Atlantic sector in Antarctica (Fig. 3g). We found that except for the Weak Monsoon interval 3, all other episodes are concurrent with warmer Antarctic temperatures.

Tiwari et al. 2021^50^ generated an upwelling record (BP/13A) from the tropical Southern Hemisphere, reconstructing the strength of upstream ISM winds. The authors focused on the difference between oxygen isotope values of surface and thermocline dwelling *Gs. ruber* and *Gr. Menardii* respectively. The reduced difference was interpreted as indicative of surface upwelling and thus, stronger ISM winds (and vice versa). From the record which spans 145 kyr, they observed a declining ISM trend from ~ 110 to 80 kyr BP. Upon comparing our record with the BP/13A reconstruction, we observe several coherent episodes of weaker monsoon variability (Fig. 3e) during the weak upwelling. These weak upwelling episodes are associated with the weaker cross-equatorial flow of winds, lowering the ISM intensity. Strong correlation between ISM and southern Indian ocean millennial scale variability was also reported from a sediment core (ABP-S4, 40° 3′ 30″ S; 48° 5′ 44″ E) raised near the Mascarene High^57^ (20°–40° S and 45°–100° E) in the mid-latitudes. Based on Mg/Ca SST reconstruction and simulated SSTs during MIS 3 to the mid-Holocene period, authors reported high (low) ISM during low (high) southern mid-latitude SSTs. SSTs in the mid-latitude Indian ocean are modulated by several processes in the Southern Hemisphere, such as the strength of the Agulhas leakage current, the strength of the AMOC and, subtropical frontal shifts. As a result, variability in these processes governs the SSTs near Mascarene High, impacting the temperature gradient with the Indian Low. Additionally, they observed a strong correlation between simulated SSTs and surface pressure gradient in the Indian ocean; during high SSTs in the mid-latitudes, low surface pressure gradient was reported. Their work also supports the hypothesis that ISM strength is modulated by the cross-equatorial flow of winds which are controlled by surface pressure gradient between the Mascarene High and the northern Arabian Sea.

We then compared our record with the relatively lower-resolution SST reconstructions based on radiolarians from core ODP 1089^51^, located in the south Atlantic subtropical front representing the strength of Agulhas leakage (Fig. 3f). Overall, we find that warmer SSTs in the Atlantic sector corresponding to stronger Agulhas leakage events are associated with weaker ISM episodes. One mechanism connecting the strength of the Agulhas leakage and ISM variability is postulated by Nair et al. 2019^25^. ISM variability is connected to the Southern Hemisphere, Mascarene High via Hadley circulation^31^. The northward expansion of Antarctic subpolar and polar fronts during the glacial/stadial stages, is related to Antarctic ice sheet growth and equatorward shifts of the Southern Hemisphere westerlies. This leads to a northward shift in Antarctic Circumpolar Current (ACC), weakening the strength of the Agulhas leakage. Subsequently, lowered transport of eddy parcels of heat flux to the south Atlantic Ocean, result in low temperatures at Site ODP 1089. However, the heat flux is redirected to the Indian Ocean via the ACC and thus inducing warmer SSTs in the higher southern latitudes during cold periods. This potentially weakens the pressure gradient between the Mascarene High and the Indian Low, which ultimately weakens the cross-equatorial flow of south-easterly winds.

Conversely, when the ACC shifts southwards, the South Equatorial Current is strengthened by intensification of the Agulhas Current and Agulhas leakage. Consequently, higher SSTs at ODP Site 1089 are observed as heat fluxes escape into the Atlantic sector. Subsequently, the ACC carries less heat towards the Indian Ocean, reducing the SST in this domain. Colder temperatures in the high latitudes of the Southern Indian Ocean increase the cross-equatorial flow of monsoonal winds, with noted increases in seasonal monsoon rainfall.

In addition to the above mechanisms, the SAM, which is also related to frontal shifts, is observed to affect the strength of the ISM^22,23^. Viswambharan and Mohankumar, 2013^22,23^ observed significant negative correlations between June SAMI and July–August rainfall. The authors found that SST warming in the Southern Hemisphere was concurrent with cooling over the Arabian sea which eventually weakened the meridional temperature gradient. Consequently, weak moisture flow during the peak monsoon period reduces precipitation over Peninsular India during periods of high SAMI. Although SAMI operates on inter-annul timescales, we postulate that similar shifts in Southern Hemisphere Westerlies operating over sub-orbital timescales may also affect ISM strength.

## Methodology

Sixteen ^230^Th ages (Table S1, Fig. S2) were measured from stalagmite using U/Th methods. The samples were analyzed by a Nu Instruments Multi-Collector Inductively Coupled Mass Spectrometer (MC-ICPMS) at the Oxford University, UK^58^. U concentrations were measured using a bracketing standard approach. Th was measured against in-house Th standards^59^. Half-lives as calculated by Cheng et al. (2013) were used for calculations^60^. The age data was further corrected for the presence of detrital Th using bulk detrital value of + 5.38E − 06 and − 4.84E − 06^61^. Five ages (Fig. S2) show reversals due to low U and relatively high ^232^Th concentrations. As a result, these ages have been discarded. The middle section of the stalagmite, between the two hiatus periods, shows high detrital content and only one age could be derived. The age model for the sample was constructed using COPRA, an interactive age model^62^. A median age model with 2000 Monte-Carlo simulations and 95% confidence interval was derived. Since only one age was obtained for the middle section, this section has been excluded from the age model. The chronology of the stalagmite on either side of this middle section was modeled independently and merged to establish a composite time series.

For stable isotope analyses of oxygen and carbon (δ^18^O and δ^13^C), 861 sub-samples were extracted from Sections 1 and  3, SI Fig. S2 using a New Wave Research Micromill, with a spatial resolution of 400 μm at the Physical Research Laboratory (PRL), Ahmedabad, India. Due to lot of detritus content, section-2, SI Fig. S2 was not attempted for detailed analytical measurements. Sub-samples weighing ~ 500 µg were reacted with 100% ortho-phosphoric acid at 72 °C in a GasBench and δ^18^O and δ^13^C values in the liberated CO_2_ were measured on a Delta-V plus IRMS at PRL. NBS-19 was used as an international standard and a laboratory standard prepared from Makrana marble with 99.9% purity was used as an internal reference. All the values are reported with respect to VPDB. The precision (1σ) of δ^18^O and δ^13^C measurements were ~ 0.06 ‰ and ~ 0.04 ‰ respectively.

Forty-seven sub-samples were milled using a dentist’s drill at a resolution of 3 mm for major and trace element analyses from section-3, SI Fig. S2. Sub-samples weighed 6–10 mg. For trace element measurement, procedure described by Eggins et al. (1997) and modified by Zhou et al. (2008) were used^63,64^. Samples were milled in a clean environment and dissolved in 2% nitric acid. Since the solution had no residue, no further processing was required, and were measured directly on an Inductively Coupled Plasma Mass Spectrometer at PRL. For internal standardization, multiple enriched isotopes of In-Ga-^209^Bi were used. A synthetic limestone standard, JLs-1 was used for calibration. Two separate dilutions were made for high weight percentages of Ca and low concentrations of Mg, Sr, Ba and Mn. The concentrations of Mg, Sr, Ba and Mn were normalized with that of Ca.

## Supplementary Information

Supplementary Information.
