# Supplementary material for: Southern hemisphere forced millennial scale Indian summer monsoon variability during the late Pleistocene
Source: Sci Rep. 2022 Jun 16;12:10136. doi: 10.1038/s41598-022-14010-6 (PMC9203564; doi:10.1038/s41598-022-14010-6)
Supplement: Supplementary file 1 — Supplementary Information. [file 41598_2022_14010_MOESM1_ESM.pdf]

Southern Hemisphere forced millennial scale Indian Summer Monsoon variability during the late Pleistocene

Supplementary material

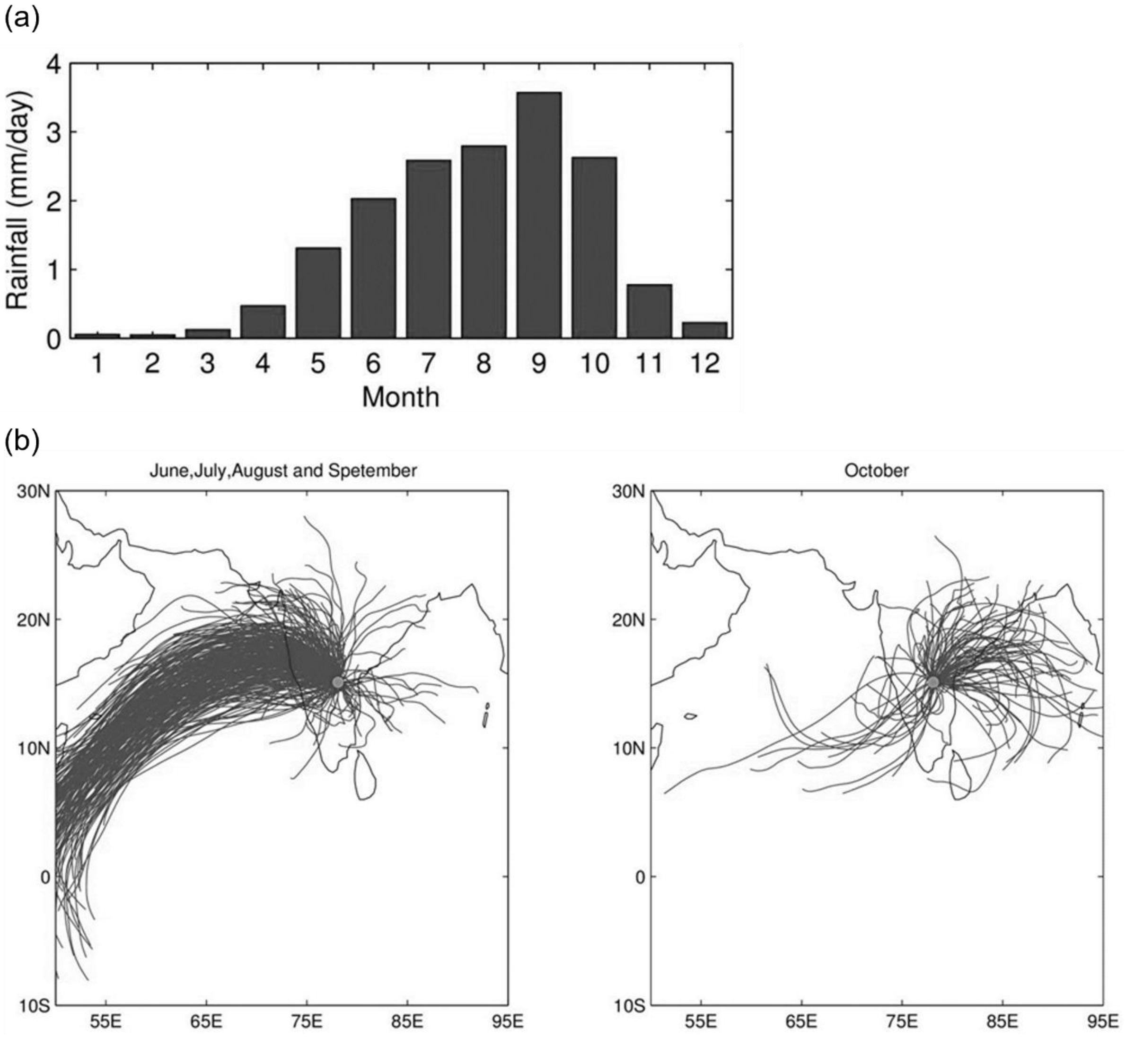

Figure S1.(a) Climatological monthly rainfall at the 0.5 × 0.5 grid over Belum cave, calculated using Asian Precipitation - Highly-Resolved Observational Data Integration Towards Evaluation (APHRODITE) data from CE1951-2007[1]. (b)Three day back trajectory estimated at 1500 m above ground level at the Belum cave location, calculated by HYSPLIT[2] using dataset from National Centre for Environmental Prediction (NCEP) reanalysis 1[3]. During a ten year period (1998-2007), all those days that receieved rain above 2mm were

considered for the analysis (JJAS-left and October-right) with the number of trajectories 384 (JJAS) and 100 (October).

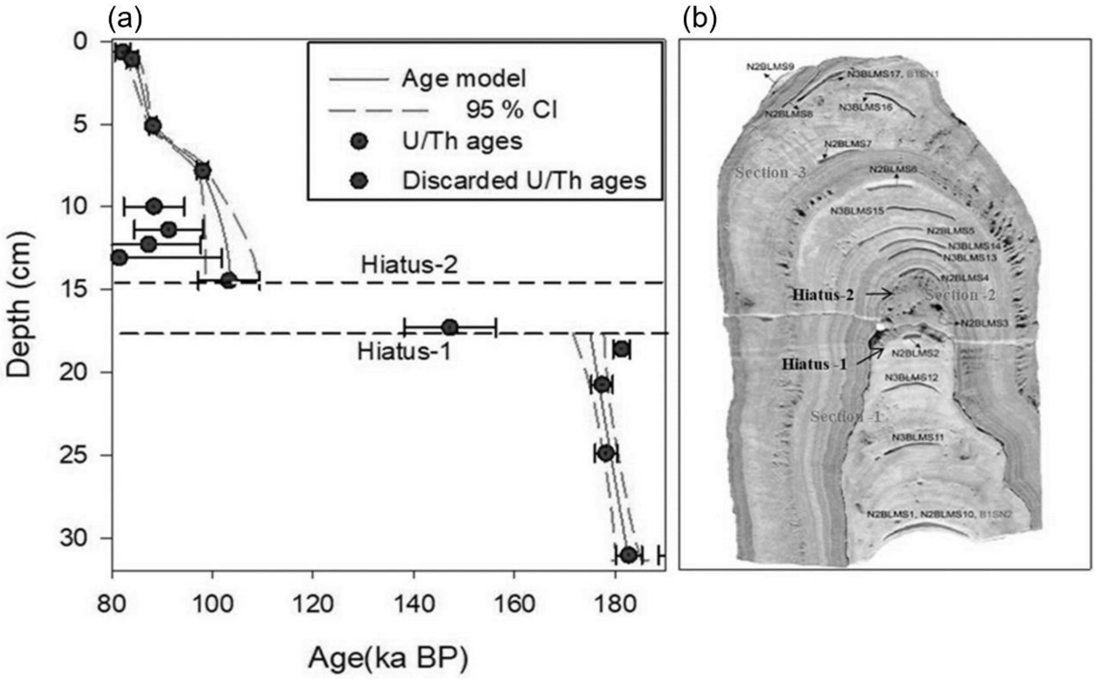

Figure S2. a) Age model reconstructed using COPRA- interactive program on MATLAB [4]. The filled blue circles are ^{230}Th ages taken for age model. Two separate age models were constructed for sections younger and older than the hiatuses and then merged. The errors reported are at 2σ level. The dashed lines show 95% confidence interval and the blue lines are the median through which the age model passes. (b) Section of the Belum stalagmite: sampling intervals for ^{230}Th ages are shown by dark curves. Section 1, (lowermost, 18-32 cm) consists of white calcitic layers, towards the end of it the boundary is marked by a prolonged hiatus1(green arrow). Section 2, (middle, 15-18 cm) is composed of layers rich in detrital particles.And hence only one ^{230}Th age could be derived. Section 3, (topmost, 0-15 cm) succeeds the former section by a brief hiatus 2 (green arrow), that covers last interglacial period. Filled red circles are U/Th ages not accounted in the age model.

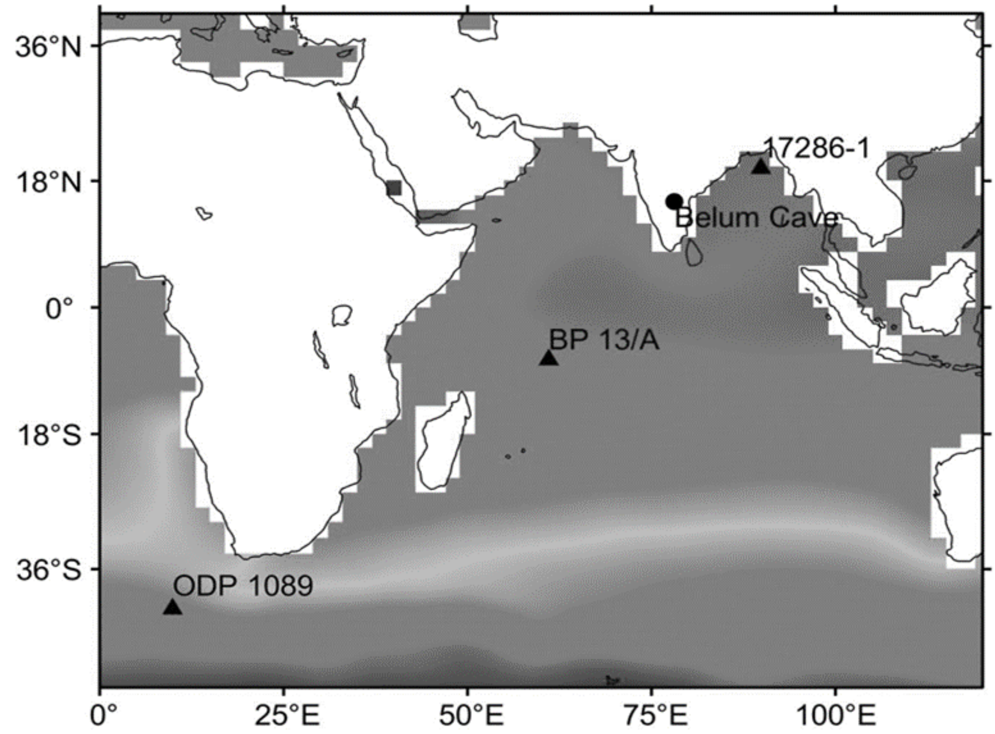

Figure S3. Sampling locations of Belum stalagmite in peninsular India (present study), sediment core SO188-17286-1[5] in Bay of Bengal, sediment core BP 13/A[6] southern tropical Indian Ocean, and, ODP 1089[7] in subantarctic Atlantic Ocean. The grey shaded area represents July -August ERSST map[8]. NOAA_ERSST_V5 data provided by the NOAA/OAR/ESRL PSL, Boulder, Colorado, USA, from their Web site at https://psl.noaa.gov/data/gridded/data.noaa.ersst.v5.html

Table S1. Uranium and Thorium isotopic compositions and ^{230}Th ages of the subsamples of Belum stalagmite. All errors are absolute 2σ values. Masses of subsample powders range from 130-200 mg (see Methodology section for the details).

Sample	^{238}U (ppb)	^{232}Th (ppt)	$^{230}Th/^{232}Th$ activity ratio	$^{230}Th/^{238}U$ activity ratio	measured (‰)	initial $\delta^{234}U$ (‰)	uncorrected Yr BP	+	-	corrected Yr BP	+	-
BLM-1(2 mm)	271.51	8.85	5.19E-04	1.03	198.24	335.81	191359	1669	1668	190420*	1776	1685
BLM-1R(2 mm)	306.99	9.75	5.27E-04	1.01	203.46	337.66	183613	2627	2460	182745	2587	2560
BLM-2(56 mm)	427.38	10.15	6.66E-04	0.97	172.04	281.60	178930	2087	1982	178222	2277	2076
BLM-3(95 mm)	424.79	10.61	6.49E-04	0.98	184.30	301.11	178150	2070	2134	177420	2124	2129
BLM-4(127 mm)§	163.62	8.34	3.36E-04	1.04	231.64	383.05	182662	1336	1186	181294	1645	1780
BLM-5(145 mm)	500.02	166.68	4.68E-05	0.95	203.26	305.16	157084	1044	1058	147201	8559	9734
BLM-6(170 mm)	396.17	90.37	5.62E-05	0.78	197.50	261.67	109933	622	558	103250	5821	6390
BLM-7(183 mm)§	82.76	59.15	1.77E-05	0.77	220.96	276.02	104313	2765	2726	81441	20375	24614
BLM-8(191 mm)§	63.60	22.54	3.48E-05	0.75	230.73	293.36	98439	3013	2969	87304	10446	10660
BLM-9(200 mm)§	97.39	25.68	4.79E-05	0.77	235.99	326.06	99099	893	1030	91289	6795	7138
BLM-10(214 mm)§	63.91	12.21	6.34E-05	0.73	243.33	310.31	93879	3206	3127	88238	6057	5790
BLM-11(228 mm)	174.28	6.76	3.35E-04	0.79	286.53	374.84	99028	521	591	98007	1112	1185
BLM-12(250 mm)	278.18	5.53	6.00E-04	0.72	269.09	342.61	88665	442	562	88089	683	767
BLM-13(280 mm)	464.44	16.634	3.25E-04	0704	274.284	344.97	84991	671	653	84023	1216	1241
BLM-14(305 mm)	272.01	13.62	2.29E-04	0.69	270.23	338.11	83534	820	769	82118	1563	1478
BLM-15(312 mm)	478.55	383.38	1.45E-05	0.70	232.76	275.03	89634	640	570	61634	24809	286938

References

1. Yatagai, A. *et al.* APHRODITE: Constructing a Long-Term Daily Gridded Precipitation Dataset for Asia Based on a Dense Network of Rain Gauges. *Bull. Am. Meteorol. Soc.* **93**, 1401–1415 (2012).

2. Stein, A. F. *et al.* NOAA's HYSPLIT Atmospheric Transport and Dispersion Modeling System. *Bull. Am. Meteorol. Soc.* **96**, 2059–2077 (2015).

3. Kalnay, E. *et al.* The NCEP/NCAR 40-Year Reanalysis Project. *Bull. Am. Meteorol. Soc.* **77**, 437–471 (1996).

4. Breitenbach, S. F. M. *et al.* COnstructing Proxy Records from Age models (COPRA). *Clim. Past* **8**, 1765–1779 (2012).

5. Lauterbach, S. *et al.* An ~130 kyr Record of Surface Water Temperature and δ18O From the Northern Bay of Bengal: Investigating the Linkage Between Heinrich Events and Weak Monsoon Intervals in Asia. *Paleoceanogr. Paleoclimatology* **35**, 1–17 (2020).

6. Tiwari, M., Kumar, V., Nagoji, S. & Mohan, R. A 145 kyr record of upstream changes in Indian monsoon circulation and its link to southern high-latitude climate. *Polar Sci.* (2021) doi:10.1016/j.polar.2021.100739.

7. Cortese, G., Abelmann, A. & Gersonde, R. A glacial warm water anomaly in the subantarctic Atlantic Ocean, near the Agulhas Retroflection. *Earth Planet. Sci. Lett.* **222**, 767–778 (2004).

8. Huang, B. *et al.* NOAA Extended Reconstructed Sea Surface Temperature (ERSST),

Version 5. *Natl. Centers Environ. Information.* (2017) doi:10.7289/V5T72FNM.
